# Deciphering T Cell Dynamics in Alzheimer's Disease Pathogenesis: Insights and Implications

**DOI:** 10.2174/011570159X350611250303044527

**Published:** 2025-03-21

**Authors:** Qiqi Yang, Yunjie Qiu, Junjun Ni, Hui Li, Hong Qing

**Affiliations:** 1 School of Life Science, Beijing Institute of Technology, Beijing, 100081, China;; 2 Department of Biology, Shenzhen MSU-BIT University, Shenzhen, 518172, China

**Keywords:** T cells, Alzheimer’s disease, peripheral immune system, microglia, tau pathology, gut microbiota

## Abstract

Neuroinflammation has emerged as a crucial factor in the pathogenesis of Alzheimer's disease (AD), paving the way for promising therapeutic interventions. Increasing evidence highlights the interplay between the peripheral immune system and the central nervous system (CNS) in driving neuroinflammation, with T lymphocytes playing a vital role in both regulatory and effector functions. Aberrant activation of T cells during the early stages of neuroinflammation perpetuates inflammatory responses by interacting with CNS glial cells and releasing pro-inflammatory mediators, such as IFN-γ, TNF-α, and IL-17. Studies have documented significant T cell activation and infiltration into the brain parenchyma in AD, contributing to disease progression. However, the specific mechanisms by which T cells mediate AD pathogenesis remain unclear. This comprehensive review synthesizes the current understanding of T cell involvement in AD pathology, emphasizing their aberrant activation, interactions with microglia, tau protein pathology, and the influence of gut microbiota. Finally, we propose potential treatment modalities for AD, highlighting the promise of T cell-based therapies currently under investigation in clinical trials. Understanding the critical role of T cells in intercellular communication and disease progression may enhance our comprehension of the pathophysiology of AD.

## 1 INTRODUCTION

Alzheimer's disease (AD) is the most prevalent form of dementia, accounting for 60-80% of all cases. It is a progressive neurodegenerative disorder that primarily affects older individuals, characterized by memory loss, cognitive impairment, behavioral changes, and difficulties in performing daily activities [1-3]. Approximately 10-15% of individuals with mild cognitive impairment transition to dementia each year, and once diagnosed with AD, they typically have an average life expectancy of 8-10 years [4]. The prevalence of AD is around 10% among individuals aged 65 and older, increasing to nearly 50% among those aged 85 and older [5]. According to the latest World Alzheimer's Report, approximately 50 million people worldwide are currently living with AD, a figure projected to rise to 82 million by 2030 and exceed 150 million by 2050 [6]. As the global population ages, the increasing incidence and prevalence of AD present a significant challenge to public health, social systems, and economic development. The primary pathological hallmarks of AD include the formation of senile plaques composed of extracellular amyloid beta (Aβ) deposits [7-10] and neurofibrillary tangles (NFTs) containing intracellular hyperphosphorylated tau protein [1, 7, 11-14]. These pathological alterations contribute to localized brain atrophy, accompanied by neuronal and synaptic loss [15, 16].

While the precise etiology of AD remains unclear, growing evidence highlights the immune system's significant role in its progression. Traditionally, the central nervous system (CNS) was regarded as immune-privileged, limiting immune cell access [17, 18]. However, recent research has revealed the involvement of immune cells, particularly T cells, in the pathophysiology of neurodegenerative diseases [10, 19-32]. T cells are crucial components of the adaptive immune system, responsible for identifying and eliminating pathogens, infected cells, and tumor cells. These cells are activated by antigen-presenting cells (APCs) and recognize antigens through the T cell receptor (TCR). Upon activation, T cells can differentiate into various subtypes, including CD4^+^ helper T cells, CD8^+^ cytotoxic T cells, and regulatory T cells (Tregs) [33]. The research data have shown that T cells present in the brains of AD patients and mouse models can cross the blood-brain barrier (BBB) through chemokines such as CCL3, CCL4, and CXCL10 [32, 34-38]. This infiltration contributes to neuroinflammation and neuronal damage by bypassing immune evasion mechanisms like the activated leukocyte cell adhesion molecule (ALCAM)-mediated homing system [39]. Dysregulated CD4^+^ and CD8^+^ T cells are implicated in exacerbating AD through various mechanisms, including the release of pro-inflammatory cytokines, direct neuronal damage, and modulation of the gut microbiome [16, 19, 20, 27, 40]. Furthermore, the association of CD3^+^ T cells with Aβ and tau pathology underscores their critical role in neuroinflammation and disease progression [23-25, 41].

In summary, T cells have a complex and critical role in the neuropathogenesis of AD. This article will provide a comprehensive review of the mechanisms by which T cells influence AD pathology, emphasizing their interactions with glial cells, tau protein pathology, and the gut microbiota. Additionally, it will explore the therapeutic potential of targeting T cells in the treatment of AD.

## 2 THE ROLE OF T CELLS IN ALZHEIMER’S DISEASE

### 2.1 T Cells

T cells are a type of lymphocyte whose development depends on the thymus. Under normal conditions, different subsets of T cells play essential roles in maintaining either effector or regulatory functions, which are crucial for preserving immune tolerance. However, in certain pathological states, subsets of effector T cells can respond to misfolded proteins and disease-specific antigens, disrupting immune tolerance and potentially leading to the proliferation of autoreactive T cells [42-44]. Within the CNS, brain-resident T cells form a distinct population of locally restricted cells that are responsible for immune surveillance [45]. T helper cell subsets, such as Th1, Th2, and Th17, play pivotal roles as mediators in neuroinflammatory diseases [46]. In AD, increased permeability of immune cells and elevated BBB permeability likely contribute to neuronal dysfunction and cognitive decline during disease progression. Emerging evidence underscores the critical regulatory role of T cells in neuroinflammation [10]. For instance, peripheral CD8^+^ T cells can infiltrate the CNS and access the cerebrospinal fluid (CSF) [28], thereby indirectly promoting neuroinflammation in AD through the secretion of pro-inflammatory mediators during their interactions with brain glial cells [10]. Additionally, self-antigens can activate autoreactive T cells, triggering pro-inflammatory and neuro-destructive immune responses [47]. In the 5×FAD mouse model, temporary depletion of Foxp3^+^ Tregs has been shown to alleviate AD pathology, indicating that Treg-mediated systemic immune suppression may play a negative role in AD pathology [2]. Recent findings suggest that targeting activated microglia and infiltrating T cells within immune hubs associated with neurodegeneration could help prevent neurodegenerative diseases and mitigate cognitive decline [13].

### 2.2 Aberrant Activation of T Cells in AD

Due to the BBB, peripheral immune cells generally have limited access to the brain. However, in AD and other pathological conditions, T cells can migrate into the brain parenchyma and actively participate in the disease process [13, 32, 34-38]. Immune system dysregulation is frequently observed in AD patients, particularly characterized by the aberrant activation of CD4^+^ T cells and CD8^+^ T cells. Activated T cells can exacerbate AD pathology through the secretion of inflammatory cytokines, direct attacks on neurons, or modulation of the microbiome [40]. Cytokines such as IFN-γ, TNF-α, and IL-17 contribute to neuroinflammatory responses in AD. They are known to promote the formation of Aβ plaques and the phosphorylation of tau protein, thereby worsening AD pathology [41].

CD3^+^ T cells have been identified in the brains of AD patients, particularly in regions exhibiting amyloidosis and tau pathology [23, 24], which contrasts with their absence in other non-AD neurodegenerative dementias [25]. In AD, there is a higher proportion of CD4^+^ and CD8^+^ T cells derived from Th17 cells, which are known for promoting inflammatory responses, compared to healthy individuals [19]. Notably, Th17 cells show significant increases, especially in the early stages of AD [20]. Additionally, studies have demonstrated heightened Aβ-specific CD4^+^ T cell responses in blood samples from AD patients [27]. In mouse models exhibiting Aβ and tau pathology, such as the 3xTg-AD and Thy1-APP751SL·HMG-PS1M146L models, there is a marked increase in CD8^+^ T cells found in CSF, hippocampal tissue, and the meninges adjacent to the hippocampus [16]. This elevation positively correlates with Braak staging [26], suggesting that T cells may contribute to AD pathology through mechanisms involving neuroinflammation or other pathways.

### 2.3 T Cells and Cognitive Dysfunction in AD

Declines in cognitive function in AD patients are closely linked to increased levels of effector memory T cells that are advanced in differentiation and contribute to mechanisms leading to neurodegeneration [13, 48]. Numerous studies consistently demonstrate a significant increase in T cells, particularly CD8^+^ lymphocytes, within the brain parenchyma of AD patients, especially in regions severely affected by aging and AD pathology [24, 26, 28-31, 45]. These T cells breach the BBB and infiltrate brain tissue, with the highest concentrations observed in areas like the hippocampus, where pathological changes are most pronounced. This infiltration strongly suggests a direct association between neuronal damage and the accumulation of T cells [16]. Further research indicates that CD8^+^ T cells exhibit a negative correlation with cognitive function in AD. Advanced techniques such as single-cell TCR sequencing have identified clonally expanded CD8^+^ T cells in the CSF of AD patients. Moreover, there is a progressive increase in the frequency of CD8^+^ effector memory T cells in the blood of AD patients, which correlates with disease progression [28]. In healthy brains, T cells are typically absent. However, studies have shown the presence of CD3^+^ T lymphocytes in postmortem brain samples from AD patients [23, 24]. Notably, the number of CD3^+^ T cells infiltrating the hippocampus is significantly higher in AD patients compared to non-demented controls, and this increase coincides with severe tau pathology in the region. In contrast, there is no significant association between these T cells and amyloid-beta (Aβ) plaques [13, 24, 37], and this infiltration phenomenon has not been observed in other neurodegenerative dementias unrelated to AD [24, 25]. In summary, the increased presence of CD3^+^ T cells in the brains of AD patients is closely associated with tau pathology, suggesting that these T cells may play a detrimental role in tau-driven neurodegeneration. Collectively, these findings highlight the significant role of T cells in the progression of AD pathology, indicating that reducing or inhibiting the activation of T cells in the peripheral immune system and their infiltration into the CNS may present a promising therapeutic approach for AD.

## 3 INTERACTION BETWEEN T CELLS AND GLIAL CELL

### 3.1 Role of Glial Cell in AD

Microglia are the resident immune cells of the CNS [49], playing a critical role in responding to injury pathogens and maintaining brain homeostasis [48]. They can be activated by infections, neuronal damage, or inflammatory stimuli, triggering either pro-inflammatory or anti-inflammatory responses [13, 50, 51]. This adaptability enables them to respond to the changing brain environment characterized by neuroinflammation and injury [52-54].

Numerous studies have consistently identified microglial activation and the resulting neuroinflammation as prominent pathological features of AD [55-58]. In various neurodegenerative diseases, activated microglia are typically found in regions where neuronal death occurs [51]. In the context of AD, microglia play a dual role: they can phagocytose and clear Aβ, thereby limiting plaque formation and local damage. However, in relation to tau pathology, microglia contribute to neuroinflammation through the non-canonical NF-kB pathway [59]. This response, triggered by neuronal injury and tau aggregation, ultimately exacerbates neurodegeneration [60]. Interestingly, in AD patients, a systemic inflammatory response is often observed preceding the onset of memory impairment, suggesting that peripheral immune activation serves as a precursor and significantly contributes to AD pathology [20-22]. Overall, neuroinflammation driven by microglial activation is widely recognized as a pivotal factor in the progression of AD [47].

Astrocytes are the most abundant cells in the brain, primarily occupying the space surrounding neurons and blood vessels. In addition to supporting the formation and maintenance of the BBB, astrocytes play a vital role in maintaining neuronal homeostasis by secreting neurotrophic factors, regulating synaptic and neurotransmitter balance, and stabilizing extracellular ion concentrations [61, 62].

Recent studies indicate that reactive astrocytes are crucial players in AD-related neuroinflammation, as these cells accumulate around Aβ deposits, triggering neuroinflammatory responses [63, 64]. When stimulated by misfolded pathogenic proteins such as Aβ and tau, astrocytes transition into a reactive state, releasing pro-inflammatory and neurotoxic factors, altering phagocytic activity, and significantly impacting synaptic connectivity [62, 65-67]. Some evidence suggests that reactive astrocytes can limit the growth of amyloid plaques and reduce associated neuronal damage [68]. However, more studies have identified an accumulation of neurotoxic C3^+^ pro-inflammatory A1-type reactive astrocytes in AD model mice and in tissues from AD patients [69].

### 3.2 Regulation of Glial Cell Activation by T Cells

**Fig. (1):**
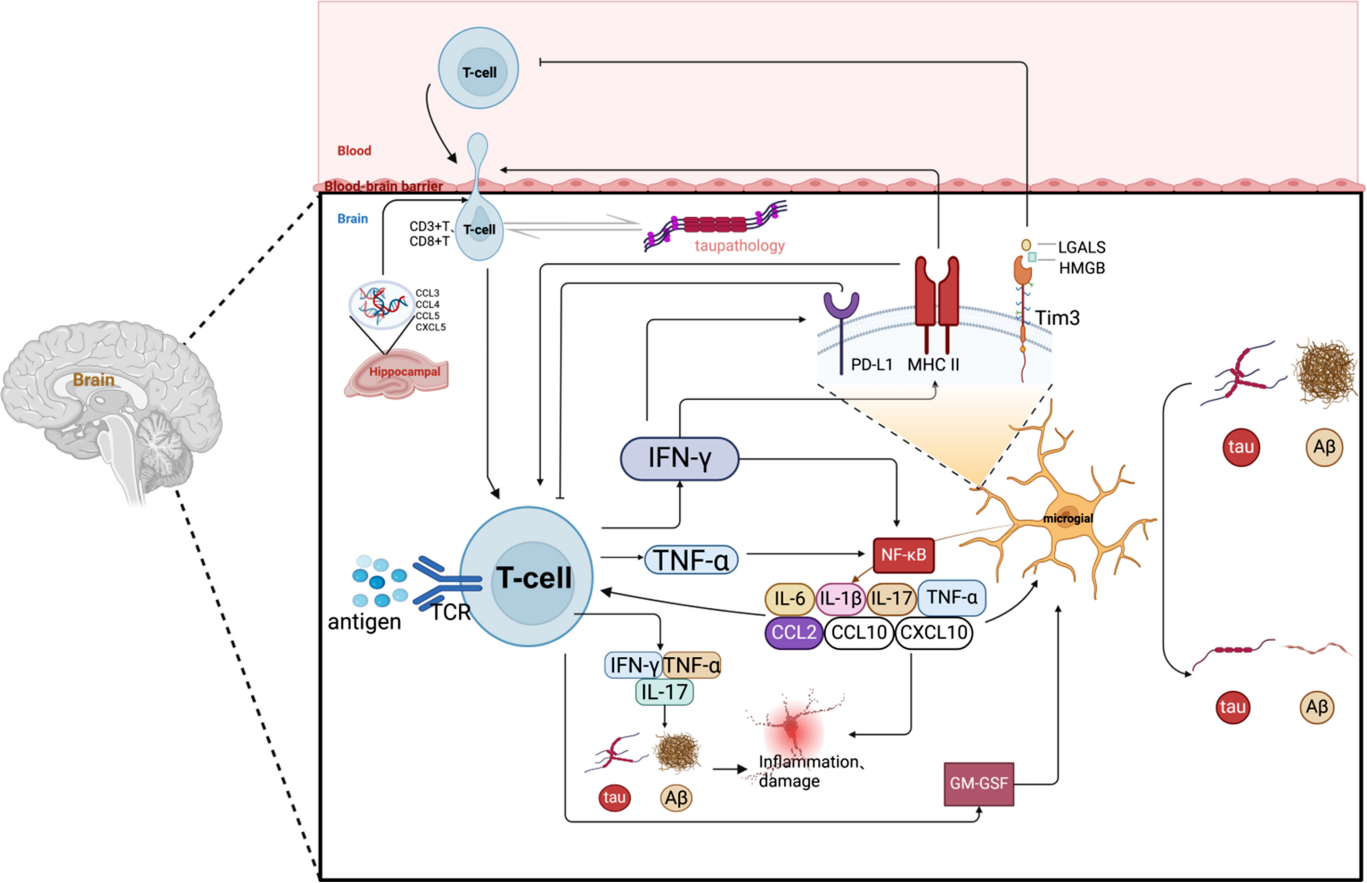
Diagram of the interaction between T cells and microglia. T cells penetrate the blood-brain barrier through chemokines secreted by microglia, such as CCL3, CCL4, CCL5, CXCL5, and CXCL10. They then secrete inflammatory cytokines IFN-γ, TNF-α, IL-1β, IL-17, and IL-6, which activate the atypical NF-κB pathway in microglia. This activation, together with GM-CSF, enhances microglial activation and upregulates MHC II. MHC II and GM-CSF further recruit and activate more T cells, inducing upregulation of PD-L1 in microglia, which can inversely inhibit T cell activity and cytokine production. Additionally, TIM-3, specifically expressed on microglia, binds to its ligands (such as Galectin-9 and HMGB1), delivering inhibitory signals that lead to T cell dysfunction or exhaustion.

T cells exert regulatory control over microglial activation through various mechanisms, including cytokine secretion, direct cell-to-cell contact, the release of regulatory molecules, and modulation of metabolic pathways (Fig. **1**).

In the human brain, CD4^+^ T cells can regulate microglial activation, potentially exacerbating neuroinflammation. The absence of CD4^+^ T cells leads to microglia maintaining a fetal-like transcriptional state, contributing to synaptic pruning defects and inducing depression-like behavior in mice [10, 70]. In microglia-astrocyte co-cultures, Th1 and Th17 cells stimulate a pro-inflammatory response in glial cells, while Th2 cells play a more regulatory role [71].

T cells activate the non-canonical NF-κB pathway in microglia by secreting cytokines such as TNF-α and IFN-γ. This pathway works in conjunction with T cell-derived granulocyte-macrophage colony-stimulating factor (GM-CSF) in neurological diseases [72]. GM-CSF enhances microglial activation, leading to increased secretion of pro-inflammatory cytokines, including TNF-α, IL-1β, and IL-6, which amplify neuroinflammation. Additionally, GM-CSF promotes the expression of MHC-II molecules and co-stimulatory molecules (*e.g*., CD80/CD86) on microglia, enhancing their antigen-presenting capabilities and further attracting and activating additional T cells. This activation creates a positive feedback loop, as these T cells secrete more GM-CSF, which amplifies neuroinflammation [72-76]. Ultimately, microglial activation represents a distinct hallmark of neuroinflammation observed in both AD patients and animal models [77].

In the TE4 (tau pathology) mouse model, the pro-inflammatory cytokine IFN-γ, produced by natural killer (NK) cells, NKT cells, and T cells, initiates the inflammatory response of microglia to injury and enhances the function of cytotoxic CD8^+^ T cells [16]. T cell immunoglobulin and mucin domain protein 3 (TIM-3), primarily expressed on activated CD4^+^ and CD8^+^ T cells, Tregs, monocytes, dendritic cells, and NK cells, is specifically upregulated in microglia within the brain. TIM-3 plays a regulatory role in microglial activity and their interaction with neurons [78]. The binding of TIM-3 to its ligands (*e.g*., Galectin-9, HMGB1) transmits inhibitory signals that can induce T-cell dysfunction or exhaustion [70, 79].

Additionally, studies have shown that Tregs help regulate the balance of reactive astrocytes in the amyloid pathology associated with AD, inhibiting the neurotoxic C3^+^ A1 phenotype while promoting the protective A2 phenotype [63]. In healthy mice, Tregs also modulate the expression of specific A1 phenotype markers [63]. In the APPswe/PS1DE9 mouse model, Treg expansion enhances GFAP expression and increases the number of astrocytes surrounding amyloid deposits [80]. However, the precise effects of Tregs on the phenotype and function of reactive astrocytes remain unclear. In the APP/PS1 mouse model, early depletion of Tregs is associated with an increase in C3^+^ reactive astrocytes around large amyloid deposits, suggesting that Tregs may play a role in suppressing the pro-inflammatory C3^+^ reactive astrocytes associated with late-stage amyloid deposition, thereby mitigating neuroinflammation and cognitive impairment [63]. Furthermore, CD4^+^ T cells in the brain can exacerbate neuroinflammation by promoting astrocyte activation, while astrocytes suppress CD8^+^ T cell activity through the expression of PD-L1 [10, 81].

### 3.3 Mechanisms of Interaction between T Cells and Glial Cell

Research indicates active interactions between microglia and infiltrating T cells [13]. In the early stages of AD, microglial activation results in increased expression of neuroinflammatory molecules such as MHC II, which present antigens to activated T cells, thereby initiating an adaptive immune response [23] and facilitating the infiltration of CD8^+^ T cells into the brain [82]. In brain regions such as the hippocampus and piriform-entorhinal cortex, reductions in microglial activity, along with decreases in the numbers of CD3^+^ and CD8^+^ T cells, are associated with reduced tau pathology in AD. This suggests that activated microglia, through the secretion of cytokines (*e.g*., IL-1β, IL-6, TNF-α) and chemokines (*e.g*., CCL2, CXCL10), can recruit and activate T cells within the brain parenchyma [13]. Additionally, IFN-γ, a cytokine produced by activated CD8^+^ T cells, significantly enhances MHC II expression and induces upregulation of PD-L1 in astrocytes [81]. During acute infection phases, microglia show sharp increases in PD-L1 expression that coincide with the infiltration of IFN-γ-secreting CD8^+^ T cells and elevated CNS TNF-α levels [81, 83]. In models of multiple sclerosis and acute viral encephalitis, microglia, together with astrocytes, utilize the PD-L1 pathway to suppress the activity and cytokine production of CD4^+^ and CD8^+^ T lymphocytes, thereby regulating their response to the virus [81, 84-86]. This mechanism, known as T-cell exhaustion, is commonly observed [87].

In neuro-pathological mechanisms, there are intricate regulatory interactions between T cells and microglia, involving both stimulatory and inhibitory effects. Understanding these interactions in AD is crucial for enhancing our comprehension of neuroinflammation. Activated T cells can amplify the neuroinflammatory response, potentially exacerbating neuronal damage and accelerating disease progression. Therefore, strategies aimed at regulating microglial activation mediated by T cells and controlling the secretion of cytokines (*e.g*., IL-1β, IL-6, TNF-α) and chemokines (*e.g*., CCL2, CXCL10) by microglia are essential for mitigating excessive neuroinflammation and managing neurodegenerative pathology, particularly in AD.

## 4 INTERACTION BETWEEN T CELLS AND TAU PATHOLOGY

### 4.1 The Important Role of Tau Pathology in AD

Tau protein, a microtubule-associated protein predominantly expressed in neurons [10, 88, 89], plays a critical role in promoting neurite elongation and stability [25, 90-94]. Research indicates that Tau protein levels in the brain tissue and CSF of AD patients are approximately 4-8 times higher than in age-matched healthy controls [95, 96]. The accumulation of Tau in various brain regions disrupts synaptic and circuit functions, contributing to learning and memory impairments [97-99]. Tau protein reduces the concentration of microtubule-associated proteins essential for microtubule polymerization in the brain [100]. In AD, abnormal phosphorylation of Tau leads to its aggregation [25], resulting in the formation of NFTs within neuronal cell bodies [16]. These NFTs disrupt synaptic function, which subsequently contributes to neuronal death [59], accelerating the progression of AD and other neurodegenerative diseases [10, 101]. Recent studies have shown that the accumulation of hyperphosphorylated Tau positively correlates with cognitive impairment, as evidenced by neuropsychological assessments in AD patients [102].

### 4.2 The Relationship between T Cells and Tau

Based on the understanding of tau pathology, recent studies have elucidated the role of T cells in this intricate interaction. Immunohistochemical analyses of brain tissue from AD patients reveal a correlation between T cell infiltration and tau protein pathology, indicating a significant relationship between the two [13, 24, 103]. In both mouse models of tauopathy and the brain parenchyma of AD patients, there is a notable presence of activated microglia and an enrichment of distinct T cell clones, particularly cytotoxic T cells [13, 24, 29-31, 103, 104]. Their abundance is strongly associated with the severity of brain atrophy. In contrast to Aβ deposition, mouse models with tau pathology manifest distinct innate and adaptive immune responses characterized by pronounced T cell activation and altered microglial activity. Notably, the depletion of either microglia or T cells effectively prevents neurodegeneration associated with tau pathology [13]. Furthermore, in THY-Tau22 mice, the analysis identifies 28 overexpressed genes in the hippocampus, including four chemokine genes: *CCL3*, *CCL4*, *CCL5*, and *CXCL5*. These genes significantly enhance the production of chemokines, promoting T-cell infiltration into the brain parenchyma [37]. Additionally, T-cell depletion has been shown to improve spatial memory in THY-Tau22 mice, emphasizing the critical role of adaptive cellular immunity in the tau-induced cognitive deficits observed in AD [37].

Among these, Th17 cells, known for secreting IL-17, are implicated in the early inflammatory processes of AD, with their levels progressively increasing as the disease advances. The proportion of Th17 cells positively correlates with total Tau and phosphorylated tau (pTau181) levels [20, 24, 103]. Studies indicate that T cell infiltration into the hippocampus plays a significant role in tau-driven pathophysiology and cognitive impairments in AD and other tauopathies [37]. Significant infiltration of CD3^+^ T cells has been observed in the hippocampal tissue of AD patients, particularly in regions associated with tau pathology, and this infiltration correlates with levels of phosphorylated tau (p-tau) but not with Aβ [13, 37, 103]. This suggests that the direct entry of T cells into the CNS may contribute to tau pathology in AD [13, 24]. The association between CD3^+^ T cells and tau pathology is particularly pronounced in the hippocampus, where hyperphosphorylation of tau leads to the formation of NFTs and subsequent neuronal loss [10, 24]. Moreover, CD8^+^ T cells show a significant correlation with the severity of tau pathology, potentially influenced by brain-derived chemokines that attract T cells and may impact BBB permeability [105]. Within the brain, CD8^+^ T cells secrete IFNγ, which can exacerbate tau pathology and neurodegeneration by enhancing inflammatory microglial signaling and antigen-presenting functions [13].

In the cortex of patients with frontotemporal dementia carrying the P301L tau mutation, infiltration of CD8^+^ T cells has been observed. Similarly, significant CD8^+^ T cell infiltration along the dorsocaudal axis of the hippocampus, where hyperphosphorylated tau protein accumulates, has been noted in tau transgenic (THY-Tau22) mice. This infiltration is particularly associated with an early chemokine response, especially involving CCL3. The density of CD8^+^ T cells in the hippocampus of THY-Tau22 mice begins to significantly increase from 7 months of age. In these transgenic mouse models of tauopathy, the infiltration of CD8^+^ T cells into the hippocampus promotes neuroinflammation and contributes to cognitive decline, independent of impacts on tau protein deposition or phosphorylation [37]. These findings highlight the critical role of T cells in neurodegenerative diseases and tau pathology, including AD.

## 5 INTERACTION BETWEEN T CELLS AND GUT MICROBIOTA

### 5.1 The Relationship Between Gut Microbiota and AD

#### 5.1.1 Microbiota-Gut-Brain

The gut microbiota plays a crucial role in regulating the bidirectional communication pathway between the intestinal tract and the brain, known as the “microbiota-gut-brain” (MGB) axis [106-110]. This axis encompasses a complex network involving neuroendocrine, metabolic, and immune pathways that are essential for host development, digestion, behavior, and immune regulation [111-115]. Research highlights how the gut microbiota influences cognitive functions such as learning and memory through mechanisms including metabolite production, modulation of inflammatory responses, and effects on neurotransmitter systems [110]. Additionally, emerging evidence suggests that the gut microbiota supports the early development of normal social and cognitive behaviors [116].

#### 5.1.2 Dysbiosis of Gut Microbiota and AD

Dysbiosis of the gut microbiota has been closely linked to several CNS disorders, including depression, Parkinson’s disease, and AD [117-121]. This dysbiosis can compromise gut barrier integrity, leading to increased gut permeability. As a result, bacterial toxins and inflammatory mediators may enter the bloodstream, promoting systemic and CNS inflammation. This inflammatory response is considered a significant contributor to the pathogenesis of AD [122-126].

Dysbiosis can trigger inflammatory responses through multiple mechanisms, including the activation of intestinal epithelial cells and immune cells, as well as the production of pro-inflammatory cytokines such as IL-1β, IL-6, and TNF-α. These cytokines can cross the BBB, affecting the CNS and fostering neuroinflammation and neurodegeneration [127, 128]. Notably, lipopolysaccharides (LPS) from gut microbes, including *Escherichia coli* and *Bacteroides fragilis*, have been detected in the brains of elderly individuals and AD patients, particularly in the hippocampal CA1 region and the neocortex [129, 130]. Moreover, alterations in gut microbiota composition have been documented in both AD patients [131-133] and AD mouse models [134-137], leading to increased gut permeability and immune activation. This systemic inflammation may compromise the integrity of the BBB, thus promoting neuroinflammation and damage that contribute to neurodegeneration. Additionally, gut inflammation in the elderly, combined with aging and poor dietary habits, is believed to play a significant role in the pathogenesis of AD [125]. Interestingly, dysbiosis of the gut microbiota has also been observed in spouses or partners of AD patients [138]. Experiments involving co-housing or oral administration of fecal matter from AD transgenic mice have shown that such exposure can induce dysbiosis in gut microbiota, tau protein phosphorylation, and cognitive impairment. Importantly, these effects can be mitigated through the oral administration of *Lactobacillus* and *Bifidobacterium* [138].

### 5.2 The Mechanisms of Interaction between T Cells and Gut Microbiota

#### 5.2.1 Regulation of Gut Microbiota by T Cells

T cells play a crucial role in the immune surveillance of microbial antigens in the gut [139-141]. CD4^+^ T cells recognize these microbial antigens through their TCRs [142], which directly influences the composition of the gut microbiota. Th17 cells, in particular, regulate microbiota structure by secreting cytokines such as IL-17A and IL-22 [143]. Furthermore, IL-22 enhances the antibacterial capacity of intestinal epithelial cells and stimulates the production of antimicrobial peptides, thereby contributing to the stability of the gut microbiota [144].

#### 5.2.2 Direct Regulation of T Cells by Gut Microbiota

Several studies have identified gut microbiota DNA in the thymus, underscoring its role in promoting thymic T cell expansion [145], regulating T cell metabolism, and modulating T cell function and differentiation [141]. Short-chain fatty acids (SCFAs), produced by the fermentation of dietary fiber by gut microbiota, influence BBB permeability and affect T cell metabolism and function through G protein-coupled receptors such as GPR43, GPR41, and GPR109A [146]. SCFAs promote glycolysis and mitochondrial respiration, enhancing the cytotoxicity of CD8^+^ T cells, which may contribute to the pathology of AD [147-150]. Components of gut microbiota, including LPS and peptidoglycans, interact with pattern recognition receptors on host cells (*e.g*., Toll-like receptors (TLRs) and nucleotide-binding oligomerization domain-like receptors (NLRs)), activating immune signaling pathways through MyD88-dependent or TRIF-dependent mechanisms [151, 152]. This activation leads to the engagement of transcription factors such as NF-κB and IRF3, promoting the production of inflammatory factors and T cell activation, which can result in inflammation and neuronal damage [153, 154].

Additionally, TLR activation enhances the maturation and activation of antigen-presenting cells (APCs), increasing the expression of major histocompatibility complex (MHC) molecules [155], thereby facilitating T cell activation and differentiation [156, 157]. NLRs can activate NF-κB and MAPK signaling pathways, further promoting inflammatory responses and T-cell regulation [158]. Metabolites of gut microbiota, such as tryptophan metabolites, engage the aryl hydrocarbon receptor (AhR) to regulate gene expression, promoting the differentiation of Tregs and Th17 cells, enhancing Treg activation while inhibiting inflammatory responses [159]. Butyrate, another metabolite produced by gut microbiota, modulates CD8^+^ T cell immune activity through ID2 [160].

#### 5.2.3 Regulation of T Cells by Gut Microbiota through the mTOR Signaling Pathway

mTOR (mammalian target of rapamycin) is a crucial signaling molecule that regulates autophagy and protein homeostasis in the brain. Abnormal activity of the mTORC1 complex has been linked to the pathogenesis of neurodegenerative diseases, including AD [161-165]. Overactivation of mTORC1 can inhibit autophagy, leading to the accumulation of misfolded proteins such as β-amyloid and tau. This accumulation subsequently results in increased production of pro-inflammatory cytokines like IL-1β, IL-6, and TNF-α, which are elevated in the brains of AD patients. These cytokines exacerbate neuroinflammation and contribute to neurodegeneration, creating a detrimental cycle that accelerates disease progression.

**Fig. (2):**
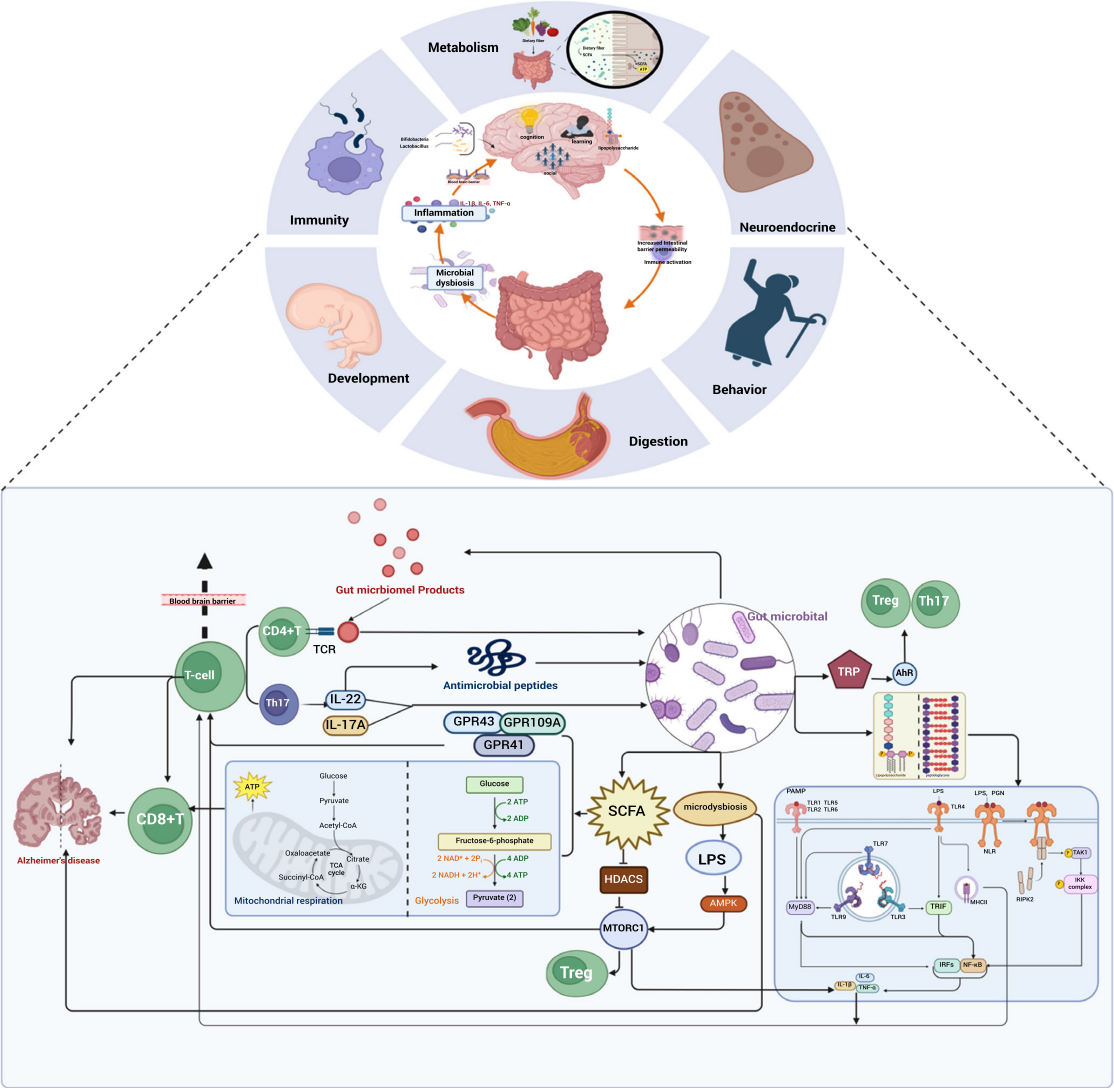
Diagram of the interaction and regulation between T cells and gut microbiota of the Microbiota-Gut-Brain. The Microbiota-Gut-Brain (MGB) Axis involves neural, endocrine, metabolic, and immune pathways and is crucial for host development, digestion, behavior, and immune regulation. Dysbiosis of the gut microbiota leads to increased production of inflammatory factors such as IL-1β, IL-6, and TNF-α, which can cross the blood-brain barrier, triggering neuroinflammation and neurodegeneration. CD4^+^ T cells recognize microbial antigens through T cell receptors (TCRs), affecting the composition of the gut microbiota. Th17 cells secrete IL-17A and IL-22 to regulate microbiota structure and promote the production of antimicrobial peptides. Short-chain fatty acids (SCFAs) enhance CD8^+^ T cell cytotoxicity through GPR43, GPR41, and GPR109A, contributing to Alzheimer's disease pathology. Lipopolysaccharides (LPS) and peptidoglycans (PGN) interact with TLRs and NLRs, activating NF-κB and MAPK signaling pathways, which promote inflammation and T cell activation. Tryptophan (TRP) metabolites regulate gene expression *via* AhR, influencing Treg and Th17 cell differentiation. SCFAs inhibit HDACs and mTORC1, reducing inflammation and promoting Treg cell differentiation. Dysbiosis increases pro-inflammatory metabolites like LPS, activating AMPK, which enhances mTORC1 activity, thereby activating T cells and further exacerbating microbiota dysbiosis.

Gut microbiota-produced SCFAs can inhibit mTORC1 by targeting histone deacetylases (HDACs) [166, 167], thereby promoting the differentiation of Tregs and reducing inflammatory responses. Conversely, dysbiosis of the gut microbiota can lead to increased levels of pro-inflammatory metabolites, such as LPS, which activate AMP-activated protein kinase (AMPK) [165] and enhance mTORC1 activity. mTORC1 regulates glycolysis and fatty acid oxidation, which activate T cells. Meanwhile, mTORC2 influences T-cell activity through the Akt signaling pathway [167]. Upon activation, T cells produce elevated levels of pro-inflammatory cytokines, such as TNF-α and IL-6 [127, 128], which can alter the composition and function of the gut microbiota, further exacerbating dysbiosis and influencing the progression of neurodegenerative diseases. Additionally, the mTOR signaling pathway plays a critical role in regulating the function of intestinal epithelial cells and immune cells, thereby maintaining the integrity of the intestinal barrier and immune homeostasis. T cells modulate their responses to gut microbiota *via* mTOR, establishing a complex feedback loop among T cells, mTOR, and gut microbiota. The interactions among these three entities promote neuroinflammation and contribute to the progression of AD [126] (Fig. **2**).

## 6 TREATMENT STRATEGIES FOR AD

We have thoroughly examined how the interaction mechanisms between T cells, microglia, tau protein pathology, and gut microbiota contribute to the progression of AD pathology. Based on our discussion, we propose several potential treatment strategies for AD, focusing on the following six aspects:

### 6.1 Regulating T Cell Function

In AD pathology, T cells frequently exhibit signs of overactivation. Using immunosuppressants to inhibit these overactive T cells or employing immunomodulators to enhance Treg function can help reduce neuroinflammation [28]. Additionally, modulating immune checkpoint inhibitory molecules such as cytotoxic T-lymphocyte antigen-4 (CTLA-4) and programmed death protein-1 (PD-1) can effectively inhibit T cell activation and suppress excessive immune responses [168-170].

Recent evidence has shown that administering low doses of interleukin-2 (IL-2) can promote the expansion of CD25^+^ Foxp3^+^ Tregs in the brain of APP/PS1∆E9 mice, further alleviating neuroinflammation [80]. In the 5xFAD-Rag2KO mouse model, transplantation of *in vitro*-expanded human Treg cells resulted in the suppression of neuroinflammation within the CNS and reduced Aβ deposition [171]. Low-dose IL-2 treatment increased CD4^+^CD25^+^Foxp3^+^ Tregs and improved cognitive function as assessed by the Y-maze test in 6-week-old APP/PS1 mice [172]. Furthermore, in a clinical trial involving AD patients aged 60-86, subcutaneous injections of IL-2 at different stages resulted in the expansion of Tregs, and cognitive improvement was observed based on the MMSE assessment [173]. It is evident that these findings contradict the preceding sections' assertion that transient depletion of Tregs can exacerbate AD pathology. These research results indicate that the role of Tregs in AD is complex and multidimensional. The contradictory results may be attributed to the use of disparate experimental models, the age of the animal model, the experimental conditions, and the targeted Tregs subtypes, among other factors.

A key factor contributing to the observed differences in outcomes may be the choice of research models. For example, while both the AD-Tg/DTR^+^ mouse model and the APP/PS1 ∆E9 mouse model are associated with AD, they exhibit distinct pathological progressions and immune responses, potentially influencing the roles of Tregs. In AD-Tg/DTR^+^ mice, cognitive function improves following the depletion of Foxp3^+^ Tregs [2]. Conversely, in APP/PS1 ∆E9 mice, cognitive function is enhanced through the expansion and activation of Tregs induced by AAV-IL-2 treatment [80]. Additionally, the stage of the disease at which Treg implication (the age of animal models) also influences disease progression and therapeutic effects. Dansokho *et al*. demonstrated the beneficial role of Tregs by targeting them during early disease stages (5-6 weeks of age in the APPPS1 model) when amyloid deposition and gliosis start developing [172]. In contrast, Baruch *et al*. investigated Tregs at an intermediate stage of Alzheimer-like pathology (4-5 months of age in the 5×FAD model) after significant amyloid-βdeposition and gliosis had developed. At this later stage, their findings suggest that Tregs have a detrimental effect, partly by reducing the choroid plexus's ability to recruit leukocytes [2]. Furthermore, the cognitive assessment methods employed in the experiments may significantly impact the results. The Morris water maze primarily evaluates spatial memory and learning capabilities, whereas the Y-maze targets short-term memory and exploratory behavior. These methodological differences may result in varied interpretations of the same treatment effects.

These factors collectively influence the effectiveness and underlying mechanisms of Tregs in AD treatment. Future research should focus on further elucidating the mechanisms of Treg function and evaluating their clinical potential, with particular attention to their efficacy and long-term sustainability in AD patients.

### 6.2 Targeted Therapy for Microglia

The overactivation of microglia in AD leads to neuroinflammation and neuronal damage. Targeting this overactivation with small molecule inhibitors or antibodies may prove effective. The proliferation and activation of microglia are closely linked to the colony-stimulating factor 1 receptor (CSF1R) [174-176]. Inhibitors of CSF1R, such as PLX3397, can attenuate microglial overactivation and proliferation, thereby reducing neuroinflammation [177-181]. However, while PLX3397 can deplete microglia, its use carries the risk of long-term adverse effects, including hepatotoxicity, nausea, vomiting, fatigue, and headaches [182-184].

Furthermore, enhancing the microglial capacity to clear Aβ plaques and tau protein pathology is vital for alleviating AD symptoms [185-187]. The triggering receptor expressed on myeloid cells 2 (TREM2) plays a critical role in microglial function, and activating the TREM2 signaling pathway can enhance the clearance capabilities of these cells, thereby mitigating AD pathology [188-191]. Agonists like AL002 have the potential to boost TREM2 signaling, promoting Aβ clearance and improving outcomes in AD [192].

### 6.3 Intervention in Tau Protein Pathology

Excessive phosphorylation of tau protein is a hallmark of AD pathology. Developing drugs that target abnormal tau phosphorylation has the potential to slow the progression of tau-related pathology. Glycogen synthase kinase-3β (GSK-3β) is a major kinase responsible for tau protein phosphorylation [193]. Inhibiting GSK-3β, for example, with the drug LY2090314, can help reduce excessive tau phosphorylation [194-196].

In addition, enhancing autophagy pathways or utilizing specific proteases to promote tau degradation can mitigate tau accumulation. Heat shock protein 90 (Hsp90) serves as a molecular chaperone that stabilizes tau protein [197]. Inhibiting Hsp90, with compounds such as 17-AAG, can facilitate the degradation of tau [198-201].

### 6.4 Regulation of Gut Microbiota

An imbalance in gut microbiota can initiate a series of pathological processes that influence AD pathology. Supplementing with probiotics or prebiotics to restore gut microbiota balance may help regulate T cell function and reduce systemic inflammatory responses [202]. Additionally, using specific antibiotics to target harmful gut bacteria or implementing dietary interventions, such as increasing fiber intake, can positively affect gut microbiota composition, influencing T cell function and inflammation levels.

Metabolites produced by gut microbiota, such as butyrate, play a crucial role in regulating the function of Tregs. Supplementing with SCFAs or their precursors can enhance the proportion of Tregs and mitigate neuroinflammation [203, 204]. Furthermore, gut microbiota can trigger systemic inflammation by affecting gut barrier permeability and activating TLR4 [205]. TLR4 antagonists, such as TAK-242, can inhibit this inflammatory response, thereby reducing its impact on the CNS [206].

### 6.5 Multi-Target Therapy

Given the complexity of AD, single-target therapies may have limited efficacy. Multi-target therapy, which integrates the aforementioned approaches, can simultaneously regulate T cells, microglia, tau protein pathology, and gut microbiota, resulting in a more comprehensive treatment effect. For example, substantial evidence suggests that impairments in the Nrf2 activation pathway in the hippocampus are closely associated with cognitive deficits and neuroinflammation [207-212].

In the treatment of AD, combining non-steroidal anti-inflammatory drugs (NSAIDs) with antioxidants such as vitamin E can effectively address both inflammation and oxidative stress [213, 214]. Additionally, curcumin may serve as a promising adjunctive treatment for AD due to its significant antioxidant and anti-inflammatory properties [215-218]. Therefore, developing small molecule drugs that target multiple pathological mechanisms, such as BACE1 inhibitors, can not only reduce Aβ production but also provide anti-inflammatory effects [219, 220]. This multi-faceted approach has the potential to enhance overall therapeutic outcomes for AD patients.

### 6.6 Biomarker-Based Personalized Therapy

By detecting specific biomarkers in AD patients, we can assess disease progression and inflammation status, paving the way for personalized treatment plans. For instance, analyzing the proportions of T cell subsets, states of microglial activation, levels of tau protein phosphorylation, and gut microbiota composition can help identify individual patients' specific pathological mechanisms and guide the selection of the most appropriate treatment strategies [14, 221].

Once the individual pathological mechanisms are identified, precise drug delivery can be achieved using nanocarriers or targeted delivery systems, enabling accurate administration of medications to affected areas. This approach can reduce side effects and enhance therapeutic efficacy [222, 223].

Through these strategies, we aim to improve our understanding and treatment of AD, slow its progression, and enhance the quality of life for patients. Future research should continue to validate the effectiveness and safety of these methods while exploring additional therapeutic targets and strategies.

## CONCLUSION AND PERSPECTIVE

This review summarizes the latest research advances regarding the interactions among T cells, microglia, tau pathology, and the gut microbiome in AD. We demonstrate that these elements form a complex network of interactions that contribute to AD pathology [10]. Microglia facilitate T cell infiltration through inflammatory responses and antigen presentation, while T cells aggravate tau pathology and neurodegeneration by secreting cytokines [13, 82]. The gut microbiome not only directly regulates T cell function but also influences both microglia and AD pathology through its metabolic products [110, 122-125, 141, 145, 224].

Despite these insights, current research encounters several limitations, including an insufficient understanding of the underlying cellular mechanisms, a lack of clinical validation, and a need for increased interdisciplinary collaboration. Future research should focus on elucidating the specific roles of T cells in AD, developing immunomodulatory therapeutic strategies that target T cells, and employing advanced techniques for comprehensive pathological analysis. Additionally, strengthening the translational research between basic science and clinical applications will provide new perspectives and methods for the early diagnosis and targeted treatment of AD. In summary, this review offers a fresh perspective for future AD research and aims to advance the development of this vital field.

## LIST OF ABBREVIATIONS

AD: Alzheimer's Disease
AhR: Aryl Hydrocarbon Receptor
ALCAM: Activated Leukocyte Cell Adhesion Molecule
AMPK: AMP-activated Protein Kinase
APCs: Antigen-presenting Cells
Aβ: Amyloid beta
BBB: Blood-brain Barrier
CCL: C-C Motif Chemokine
CD: Cluster of Differentiation
CNS: Central Nervous System
CSF: Cerebrospinal Fluid
CXCL: C-X-C Motif Chemokine Ligand
GM-CSF: Granulocyte-macrophage Colony-stimulating Factor
HDAC: Histone Deacetylase
IFN-γ: Interferon-gamma
IL: Interleukin
LPS: Lipopolysaccharide
MGB: Microbiota-gut-brain
MHC: Major Histocompatibility Complex
MMSE: Mini-mental State Examination
mTOR: Mammalian Target of Rapamycin
NF-κB: Nuclear Factor Kappa-light-chain-enhancer of Activated B Cells
NFT: Neurofibrillary Tangle
NLR: NOD-Like Receptor
PD-L1: Programmed Death-Ligand 1
SCFA: Short-chain Fatty Acid
TCR: T Cell Receptor
Th: T Helper
TIM-3: T Cell Immunoglobulin and Mucin-domain containing-3
TLR: Toll-Like Receptor
TNF-α: Tumor Necrosis Factor-alpha
Treg: Regulatory T Cell
